# Optimal swimming strategies and behavioral plasticity of oceanic whitetip sharks

**DOI:** 10.1038/s41598-017-18608-z

**Published:** 2018-01-11

**Authors:** Yannis P. Papastamatiou, Gil Iosilevskii, Vianey Leos-Barajas, Edd J. Brooks, Lucy A. Howey, Demian D. Chapman, Yuuki Y. Watanabe

**Affiliations:** 1 Department of Biological Science, Florida International University, Florida, USA; 2 Faculty of Aerospace Engineering, Technion, Haifa, Israel; 3 Department of Statistics, Iowa State University, Iowa, USA; 4 Shark Research and Conservation Program, Cape Eleuthera Institute, Eleuthera, Bahamas; 5 Microwave Telemetry Inc., Maryland, USA; 6 National Institute of Polar Research, Tachikawa, Tokyo Japan; 7 Department of Polar Science, SOKENDAI (The Graduate University for Advanced Studies), Tachikawa, Tokyo Japan

## Abstract

Animal behavior should optimize the difference between the energy they gain from prey and the energy they spend searching for prey. This is all the more critical for predators occupying the pelagic environment, as prey is sparse and patchily distributed. We theoretically derive two canonical swimming strategies for pelagic predators, that maximize their energy surplus while foraging. They predict that while searching, a pelagic predator should maintain small dive angles, swim at speeds near those that minimize the cost of transport, and maintain constant speed throughout the dive. Using biologging sensors, we show that oceanic whitetip shark (*Carcharhinus longimanus*) behavior matches these predictions. We estimate that daily energy requirements of an adult shark can be met by consuming approximately 1–1.5 kg of prey (1.5% body mass) per day; shark-borne video footage shows a shark encountering potential prey numbers exceeding that amount. Oceanic whitetip sharks showed incredible plasticity in their behavioral strategies, ranging from short low-energy bursts on descents, to high-speed vertical surface breaches from considerable depth. Oceanic whitetips live a life of energy speculation with minimization, very different to those of tunas and billfish.

## Introduction

Sparse and patchily distributed prey make the pelagic environment the marine equivalent of the desert, yet some of the largest predators spend their entire lives in this ecosystem. To do so they must maintain a surplus between the energy they gain from prey *E*_+_ and the energetic costs associated with searching for prey *E*_−_^1^.

Energy gained from prey can be expressed as

1 $${E}_{+}={e}_{T}T+{e}_{X}X,$$

where *X* is the distance covered by the predator during time *T*, whereas *e*_*T*_ and *e*_*X*_ are coefficients reflecting the prey density and mobility, as well as the probability of prey capture. The first term in (1) reflects the probability of prey arriving at the predators location without the predator moving, while the second reflects the probability of the predator finding prey through active searching. When the average swim speed of the predator

2 $$\langle {v}_{x}\rangle =X/T$$

is much greater than the average speed of its prey, *e*_*T*_ becomes irrelevant.

The energy spent searching for prey is

3 $${E}_{-}=T\langle P\rangle ,$$

where

4 $$P(t)={P}_{0}(\tau (t))+\frac{1}{\eta }v(t)F(t)$$

is the (instantaneous) routine metabolic rate, and the angular brackets denote an average with respect to time, so that

5 $$\langle P\rangle =\frac{1}{T}{\int }_{0}^{T}\,P(t){\rm{d}}t.$$

The routine metabolic rate *P* includes the standard metabolic rate *P*_0_, which is a function of body temperature *τ*, and the metabolic cost of swimming *vF*/*η*. *F* is the hydrodynamic thrust, *v* is the swimming speed, *η* is the chemo-mechanical propulsion efficiency.

Combining (1) and (3) together, the energy surplus of a predator is

6 $${\rm{\Delta }}E={E}_{+}-{E}_{-}=T({e}_{X}\langle {v}_{x}\rangle +{e}_{T}-\langle P\rangle )=X({e}_{X}+{e}_{T}/\langle {v}_{x}\rangle -C),$$

where

7 $$C=\frac{{E}_{-}}{X}=\frac{\langle P\rangle }{\langle {v}_{x}\rangle }$$

is the cost of transport (energy spent per unit distance moved, COT). We would expect swimming strategies that result in Δ*E *≥ 0. Nonetheless, there are two canonical swimming strategies: one that maximizes the energy surplus per unit distance, *e*_*X*_ + *e*_*T*_/〈*v*_*x*_〉 − *C*, and another that maximizes the energy surplus per unit time, *e*_*X*_〈*v*_*x*_〉 + *e*_*T*_ − 〈*P*〉. By using the first strategy, a fast-moving predator (with $\langle {v}_{x}\rangle {e}_{X}\gg {e}_{T}$) will maximize the energy gained from a given volume of water, whereas the second strategy will maximize the rate of this gain (but with a smaller yield). The two strategies become the same when Δ*E* → + 0 (i.e. when the energy surplus is very small).

Teleost pelagic predators (e.g. tuna) have elevated metabolic rates, increased gill surface areas, and muscle biochemistry enabling rapid recovery from exercise, all of which allow a decisive performance advantage over their prey^2–4^. However, notwithstanding their efficient propulsion (thunniform swimming gait, lunate caudal fin) and improved hydrodynamic shape (fusiform body, retractable fins), they pay a high energetic price to find prey. Their evolutionary design stresses maximization of the energy gained *e*_*X*_〈*v*_*x*_〉 + *e*_*T*_ over minimization of the energy spent 〈*P*〉, leading to a life of high-energy turnover with large Δ*E*, and consequently, a rapid growth rate^2,5^. We hypothesize that they swim so as to maximize the energy surplus per unit time, *e*_*X*_〈*v*_*x*_〉 + *e*_*T*_ − 〈*P*〉.

Pelagic ectothermic sharks (e.g. oceanic whitetip, blue sharks) likely have lower metabolic rates^6^, and lack the morphological and biochemical adaptations needed for rapid recovery from exercise. As such, they may have a less decisive performance advantage over prey, but may benefit from expending less energy to find prey. Their evolutionary design stresses minimization of energy spent over maximization of energy gained, and they likely live a life of low energy turnover with small energy surplus, and consequently, slow growth rates^7^. We hypothesize that they swim so as to maximize either the energy surplus per unit time or energy surplus per unit distance – if the energy surplus is small then both strategies should be practically the same.

First, we theoretically derive the optimal swimming strategies that pelagic predators should use if they aim to maximize the energy surplus per unit distance, or per unit time, respectively. We then use biologging sensors (speed/acceleration/depth/video) to see if the behavior of a representative ectothermic pelagic predator, the oceanic whitetip shark (*Carcharhinus longimanus*), matches these predictions. Oceanic whitetip sharks spend almost their entire lifecycle in pelagic waters, have slow growth rates, and can make predictable seasonal migrations^8^. We predict that their behavior aims to maximize their energy surplus.

## Results

### Theoretical analysis

The two canonical strategies are formally derived in the Methods. They can be summarized as follows:

#### Energy surplus per unit distance

1. For a given *e*_*T*_ and *e*_*X*_, the energy surplus per unit distance of an ectothermic predator is maximized by swimming at constant speed and depth, as deep (cold) as feasible. When *e*_*T*_ → 0, the optimal swimming speed is that which minimizes the COT. This speed decreases in relation to standard metabolic rate, and hence the animal should swim slower as body temperature decreases. An increase in *e*_*T*_ has the same effect as a reduction in the standard metabolic rate, and hence a slow moving predator (whose speed is comparable with the average speed of its prey) should swim slower than it would to minimize the COT.

2. When *e*_*T*_ → 0 and when swimming optimally, the average active metabolic rate will be in excess of 1.5 times the standard metabolic rate (see equation (38)). The excess depends on the morphology of the fins and buoyancy, increasing with the sinking factor (animal is less buoyant) and decreasing with increasing span of the pectoral fins.

3. Assuming the animal has to swim with a series of alternating dives (‘yo-yo’ diving), the optimal strategy would be diving at shallow angles relative to the horizon, with constant speed throughout the dive, and at the same speed that would minimize the COT if swimming at constant depth. The (relative) effect of diving on the COT is small - approximately half the variance of the dive angle (see equation (39)). The (relative) effect of possible variations in speed along the course is approximately the same as the variance of the (relative) speed fluctuations (see equation (39)).

#### Energy surplus per unit time

4. The above points apply to this swimming strategy as well, except swimming speed should be higher than the speed that maximizes the energy surplus per unit distance. The difference in speed is proportional to the energy surplus (there would be no difference if there is no energy surplus), and therefore swim speed should increase with prey density.

### Field data

#### Dive behavior and swim speeds

**Figure 1:**
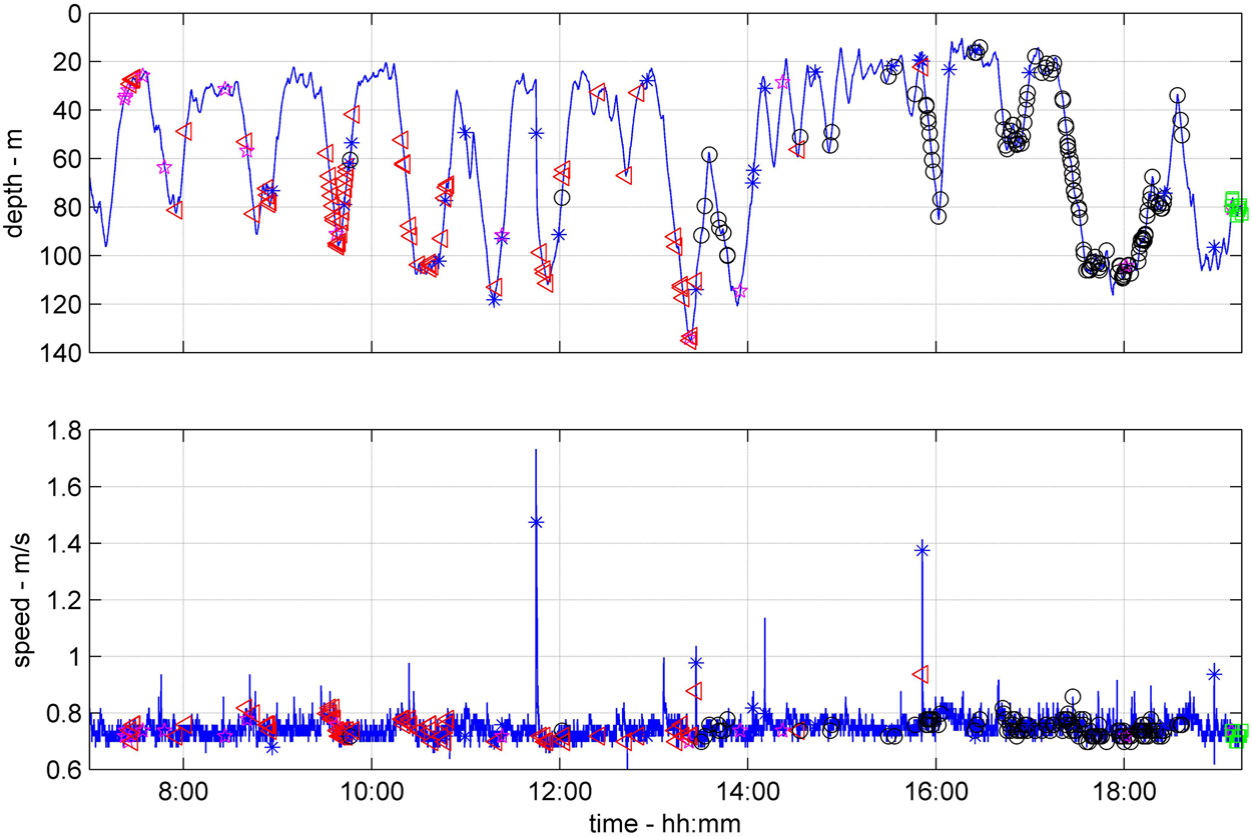
Depth (top) and speed (bottom) of an oceanic whitetip shark (OWT3). Symbols identify when the shark encountered potential prey items as determined from a dorsal fin mounted video camera: fish in general (red triangles), mackerel scad (circles), squid (squares). Possible hunting events identified in the video footage by erratic behavior or by increased activity, have been marked by asterisks. The shark performed yo-yo dives at practically constant swim speed.

**Figure 2:**
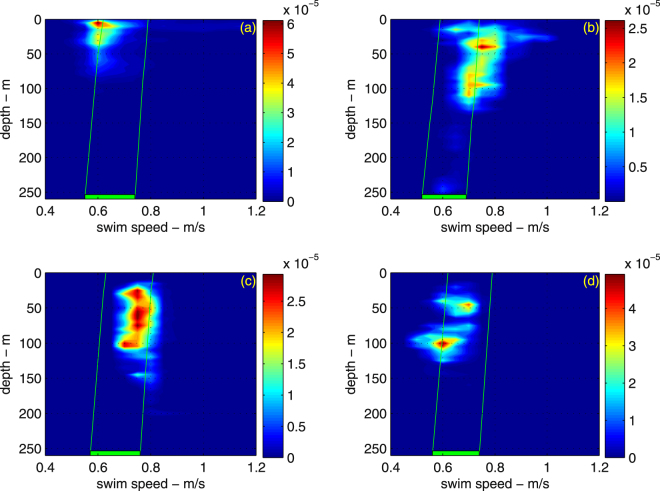
Density distribution of shark’s swim speeds with depth (panels (a–d) correspond to sharks OWT1-OWT4). The density is normalized so that the integral over the map area yields unity. The horizontal green bar represents the predicted swim speeds that minimize the cost of transport, including error bracketing. The green lines are the predicted change in optimal swim speed with depth (due to changes in water temperature).

**Figure 3:**
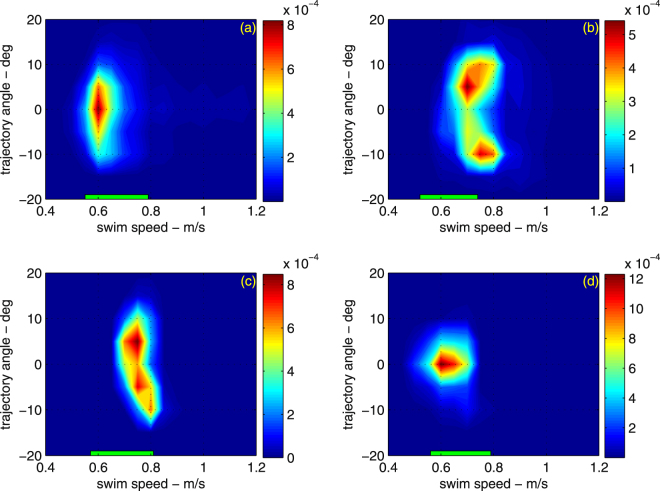
Density distribution of shark swim speeds with dive angles relative to the horizon (panels (a–d) correspond to sharks OWT1-OWT4). The density is normalized so that the integral over the map area yields unity. The horizontal green bar is the predicted optimal swimming speed that minimizes the cost of transport, including error bracketing.

We successfully recovered data-logger packages from four oceanic whitetip sharks, providing a total of 9 days of data (Table 1). All four sharks performed a series of continuous bounce (yo-yo) dives within the upper 250 m of the water column, swimming at average speeds of 0.6–0.7 m/s (Figs 1–3), but with occasional high-pitch bursts up to 4.6 m/s (Supplementary Figs S1 and S2). All four sharks used shallow dive angles, maintained near constant speed between descent and ascent portions of the dive, and swam within the speed range that should have minimized their COT (Figs 1–3). The smallest shark (OWT2) was also the fastest relative to its respective speed range. All sharks reduced their average swimming speed with depth, consistent with the animals maintaining swimming performance as the water temperature decreased (Fig. 2).

**Table 1:** Deployment information for oceanic whitetip sharks tagged in the Bahamas. Sensors: A-acceleration, H-magnetic heading, D-depth, S-speed, T-water temperature, V-video.

Deployment date	Shark	Total length (m)	Pre-caudal length (m)	Sex	Sensors	Duration (h)
May 8, 2013	OWT1	2.71	1.96	F	A, D, S, T, H	72
May 1, 2014	OWT2	2.2	1.5	F	A, D, S, T	22
May 3, 2014	OWT3	2.85	2.09	M	A, D, S, T, V	48
May 5, 2014	OWT4	2.62	1.9	F	A, D, S, T, V	43

Video footage was obtained from two sharks (OWT3 and OWT4) for a total of 16 hours. Video was used to verify speed and acceleration measurements (by confirming that the segments of increased lateral motion seen on the video corresponded to the segments of increased swimming speed and lateral acceleration) and demonstrated that sharks encountered potential prey, such as mackerel scad (potentially *Decapterus macarellus*) and squid, at depths between 20–100 m (Fig. 1). OWT3 encountered a squid patch in excess of 10 individuals at 100 m depth (Supplementary video). The encounter rate with potential prey was 0–29 within a 30 minute block (median 4) for OWT3, and 0–2 within a 30 minute block (median 1) for OWT4. No other sharks were seen in any video footage.

#### Energetic costs and hunting tactics

**Figure 4:**
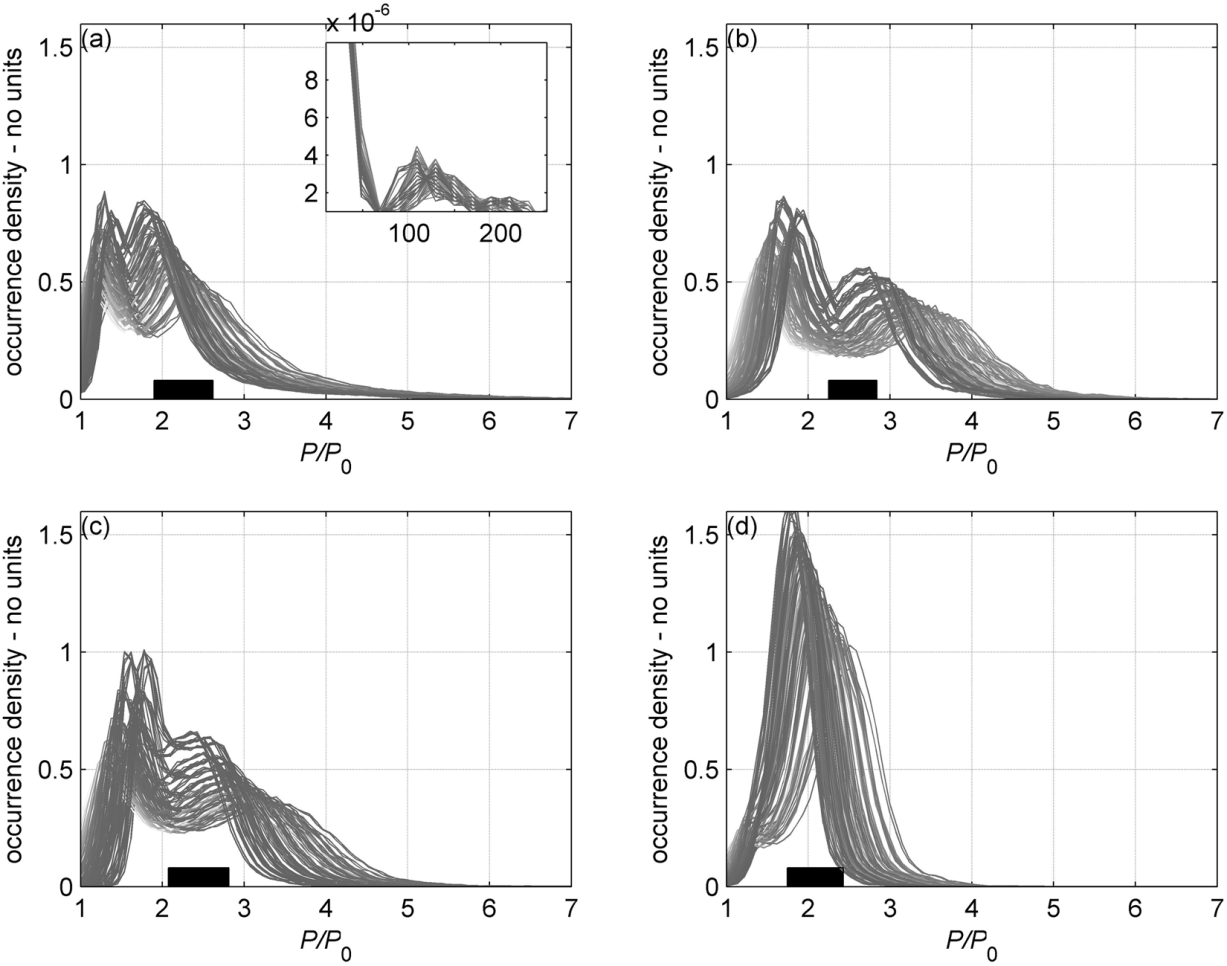
Estimated routine metabolic rates of oceanic white sharks OWT1-OWT4 (**a**–**d**). Active metabolic rate (P) is given relative to standard metabolic rate (P_0_). Curves represent the relative amount of time sharks spent at a certain metabolic rate. Each curve signifies varying bracketed errors on morphometrics. A black rectangle at the bottom of each figure represents the average routine metabolic rates. The inset in (**a**) shows the metabolic rate of OWT1 during breaching events, which exceeded the average rate by almost two orders of magnitude. The double ‘hump’ shape of the curves manifests descent and ascent phases of the shallow dives.

Based on the swimming speeds, we estimate an average routine metabolic rate 2.5 times the standard one (Fig. 4). The standard metabolic rate of a 100 kg shark at 26 °C is estimated at 15 Kcal/hour (Supplementary Table S3). When calculated cumulatively throughout the diel cycle, it yields a daily energetic cost of approximately 900 Kcal. Assuming the energetic costs of excretion and egestion are 30% ingested energy, then sharks must consume approximately 1300 kcal/day, which is the equivalent of approximately 1.5 kg prey (e.g. squid), or 1–1.5% of the shark’s body weight per day. Energy requirements were noticeably lower during the descents vs ascents as sharks are negatively buoyant, and accelerometers showed much lower activity during the descent portions of dives (Fig. 4).

**Figure 5:**
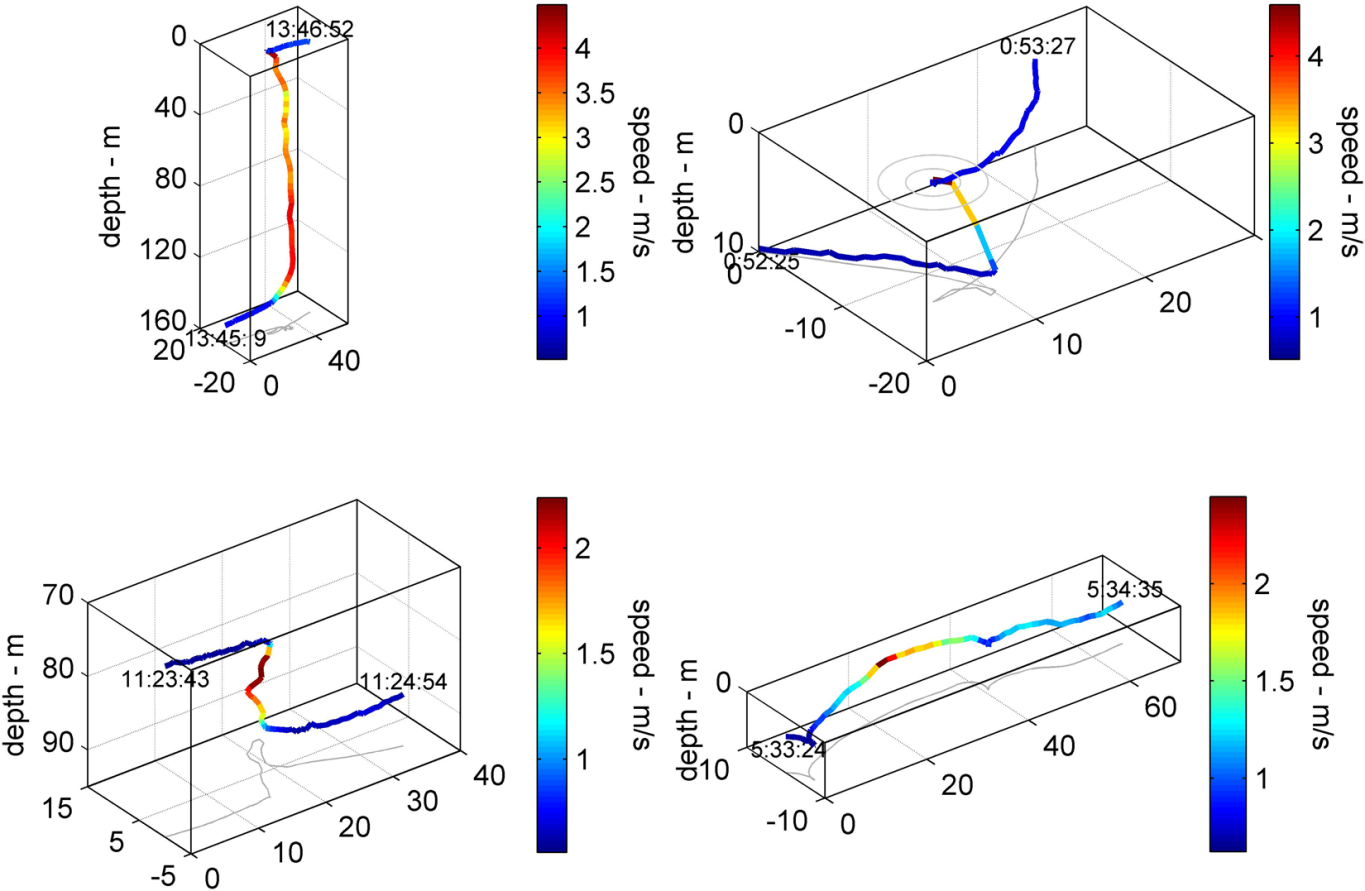
Hunting plasticity in shark OWT1. Dives are color coded by swim speed. All plates have been oriented so that the shark swims toward the upper right corner. Start- and end-times appear next to the respective points. The top row of panels shows upward bursts with possible surface breaching (circles on surface); the bottom row shows a downward burst and a long horizontal chase.

All four sharks initiated downward high-speed bursts (Fig. 5, Supplementary Fig. S2). However, shark OWT1 either behaved similarly, or performed remarkable high-speed vertical ascents (11 in total). Some of these resulted in breaches where the shark cleared the water surface (at least 7). Incredibly, one of these daytime breaches was initiated from a depth of 160 meters with a vertical ascent at 4 m/sec before the animal cleared the surface (Fig. 5). Using equation (12), we estimate that this 40-sec breach is energetically equivalent to 50 min of normal swimming (Fig. 4a). 5 of the 7 surface breaches occurred during a moonless night (on May 8, 2013).

## Discussion

Our model provides predictions as to how pelagic predators should behave in terms of diving angles and swim speeds, in order to maximize their energy surplus. Unconstrained by the necessity to search the water column vertically, the optimal strategy would be to swim at constant depth and speed, as deep (cold) as possible. Yet, almost all pelagic predators swim up-and-down, highlighting that yo-yo diving is a necessity for their survival^8–11^. Under the constraint that a predator has to search the water column vertically, the swimming strategy that maximizes the energy surplus (either per unit time or per unit distance) is to dive at small angles, and maintain almost constant speed throughout the dive. This optimal swim speed would be almost the same as the speed that would have minimized the cost of transport (COT) when swimming at constant depth – with subtle variations. It will be smaller if the speed of the predator is comparable to the average speed of its prey, and larger if the goal of the predator is to maximize the energy surplus per unit time, increasing with prey density. Indirect support for this conjecture comes from the behavior of blue sharks (*Prionace glauca*), another ectothermic pelagic predator. These sharks were consistently observed swimming at 0.3–0.4 m/s^11^, significantly slower than the speed (0.5 m/s) that would have minimized their COT^12^. However, at 0.3–0.4 m/s their swimming speed is comparable with the average speed of small prey, and hence slow swimming is consistent with an optimal swimming strategy (when *e*_*T*_ > 0).

The four tagged oceanic whitetip sharks used small dive angles, their swimming speed was practically constant throughout the dive, and within the range that would have minimized their COT if they were swimming at constant depth. There was variability in individual speeds, and we did not directly measure the buoyancy of each individual (although we included error in buoyancy estimates for our speed predictions). However, for the two sharks where video was simultaneously recorded, the individual that was swimming at the faster range of predicted speeds (OWT3) had prey encounter rates almost four times those of OWT4, which swam at the lower range of speeds, as would be predicted by our model (optimal swim speed increases with prey density). Combined, these results suggest that oceanic whitetip sharks are likely maximizing their energy surplus, and possibly maximizing their energy surplus per unit time. Previous analysis of diving behavior in whale sharks also suggested that sharks were diving at small angles so as to reduce their COT^9^.

Our estimate of shark energy costs also suggests that oceanic whitetip sharks have low energy requirements and it is likely that low prey numbers are needed to meet maintenance rations. Assuming sharks are consuming squid (an important prey item^13^), then they would only need to consume approximately 1.5% BW daily, with a video equipped shark encountering squid patches far in excess of this requirement. Of course sharks must also catch prey they encounter, but nonetheless we show that encounter rates are far in excess of the minimum numbers they need. In addition to these behavioral strategies, oceanic whitetips have morphological adaptations to optimize their swimming performance. Their broad pectoral fins make their swimming performance less sensitive to changes in buoyancy, which would be advantageous if the animal does go a long period without finding food (losing buoyancy^12^).

A likely function of yo-yo dive behavior is searching for prey, as previously suggested for tiger sharks^10^. Further evidence comes from the oceanic whitetip sharks with cameras, as potential prey were most commonly seen during the dive (and not when shallow) and at the apex of the dive, which was also when bursts of speed were common. Assuming bursts in swim speed are associated with foraging (and the video analysis seems to confirm this assumption – see Fig. 1), then the primary hunting tactic oceanic whitetip sharks use to catch prey were short bursts of speed while descending, which fits well with an energy minimizing strategy. However, one of the four tracked sharks (OWT1) displayed remarkable behavioral plasticity being also capable of performing high-speed ascents at speeds in excess of 4 m/sec. These included vertical ascents initiated at 160 m depth and ended with the shark breaching the surface. The energetic cost of a long breach are significant – a 40 sec breach at 4 m/s is energetically equivalent to 50 min of swimming at the average cruising speed. While we cannot definitively address the cause of the behavioral plasticity, we suggest it is related to prey type. A stable isotope study indicated that oceanic whitetip diet is dominated by lower trophic level prey (e.g. squid) over the course of the year, but higher trophic level prey (e.g., marlin, tuna, dolphin fish) are regularly consumed during their residency period at Cat Island^13^. We hypothesize that the high-speed surface ambush is used for these larger pelagic fish. However, we also acknowledge that surface breaching has been seen in sharks that do not appear to be foraging, and there may be other functions of this behavior. Regardless, different behavioral strategies may vary widely in their energetic costs. Large predators from both terrestrial and marine systems show plasticity in hunting strategies at the individual level, but only recently are we starting to identify these and quantify the energetic costs^5,14,15^.

Ectothermic sharks in general have lower standard metabolic rates than endothermic pelagic teleosts (tunas, billfish)^3,6,16^. The high metabolic rates and warm muscles of tunas and other endothermic fish enable them to have a large aerobic scope and swim at faster cruising speeds, while specialized muscle biochemistry allows rapid recovery from exercise^2,3,17^. When combined with their fast rates of digestion, tunas and billfish are adapted for rapidly finding, digesting and assimilating prey, albeit at considerable energetic cost^3,5,18^. Hence, they gamble a lifestyle of high costs for a high rate of return (possibly maximizing the energy surplus per unit time), which leads to the fast growth rates of surviving individuals, termed ‘energy speculation with maximization’^3^. Oceanic whitetip sharks appear more suited to an ‘energy speculation with minimization’ strategy; lower risk for lower return, which is also displayed in their slow growth rates^7,19^.

## Methods

### Experimental guidelines

Research and animal tagging was conducted under Cape Eleuthera Institute research permit (MAF/FIS/17 & MAF/FIS/34) issued by the Bahamian Department of Marine Resources under accordance with Cape Eleuthera Institute animal care protocols.

### Tagging

Fieldwork was conducted at Cat Island, Bahamas (N 24.2133, W 75.3645), in 2013 and 2014, where relatively high densities of oceanic whitetip sharks can be found from April to June. Sharks were caught on hook and line, restrained alongside the boat and morphometrics (pectoral fins width, length and span, total and pre-caudal lengths) were measured in some individuals. Seven sharks were tagged with multi-sensor data-loggers, attached to the dorsal fin via a tie-wrap threaded through two small holes. Data loggers (PD3GT, 21 × 15 mm, 60 g, Little Leonardo, Tokyo, Japan) measured 3D acceleration (32 Hz for one shark, and 16 Hz for the others), swim speed, depth and water temperature (1 Hz) and were combined with an HD video camera (DVL500, 640 × 480 pixels at 30 frames/second, 5–11 h recording duration, Little Leonardo, Tokyo, Japan). Video cameras were programmed to turn on and start recording at 07:00 the day after the animal was released. One shark (OWT1) was fit with a 3MPD3GT data-logger (26 × 175 mm, 135 g, Little Leonardo), which in addition to the sensors above, also includes a magnetometer (1 Hz). At a pre-programmed time (1–4 days) the package released from the fin and floated to the surface; embedded VHF and satellite transmitters (SPOT 5, Wildlife Computers) facilitated the retrieval of the devices.

### Data analysis

The speed sensor contains a propeller, and rotations were converted to swim speed using the calibration equation described in^20^. The resolution of the speed and depth measurements was 0.02 m/s and 0.1 m, respectively. The first 10 hours of data from each shark were discarded to remove any periods associated with stress of capture. The vertical components of velocity and acceleration were obtained by differentiating a running parabolic fit of depth; axial acceleration was obtained by differentiating a running parabolic fit of speed. In both cases, the fitting window was 11 points, 5 points on each side. If the derivative of depth with respect to time exceeded the directly measured swimming speed, the vertical velocity component was set equal to the swimming speed. Dive angle was obtained from the vertical velocity and the swimming speed. Its derivative was found either by differentiating the respective parabolic fit, or by using the second derivative of depth and the first derivative of speed.

Dead reckoning using magnetometer, depth and speed measurements was used to generate a 3D track of shark OWT1. The combination of speed and vertical velocity furnished the horizontal velocity component; the combination of the horizontal velocity component and the magnetic heading furnished the North and East velocity components; their respective integrals yielded the location; depth was measured directly. Finally, we used video camera footage to estimate encounter rates with potential prey. The definition of ‘potential prey’ included fish and invertebrates (except scyphozoans), but excluded pilot fish, which accompanied the sharks.

### Theoretical analysis

Adopting the hydrodynamic model of^12^ and the general approach of^21^, we derived two canonical swimming strategies of a yo-yo-diving-negatively-buoyant shark – one that maximizes the energy surplus per unit distance and another that maximizes the energy surplus per unit time. The models predict the dive angle, swimming speed, and the associated routine metabolic rate and cost of transport. These predictions depend on span and area of the pectoral fins, submerged weight, drag coefficient and the standard metabolic rate, which is also a function of body mass and temperature. These parameters were estimated using statistical regressions and actual measurements taken from the field (see Supplementary Notes). All morphological parameters (body mass, span and chord of the pectoral fins) were bracketed ±10% about the nominal values to account for uncertainty of the estimation methods; parasite drag coefficient was bracketed between 100% and 125%, the sinking factor was bracketed between 0.025 and 0.05.

### Optimal swimming strategies

#### Formulation of the problem

Consider a predator that forages for time *T*, covering horizontal distance *X*. It is implicitly assumed that *T* and *X* are large compared with tailbeat period and stride length, respectively. It is also assumed that either *X* or *T* are given, but not both. The predator’s energy surplus Δ*E* over *T*, which is the difference between the energy gained from prey and energy spent searching for it, is given by equation (6). We seek two canonical swimming strategies, manifested in speed *v*(·) and depth *h*(·) schedules over (0,*T*), that maximize the energy surplus per unit distance Δ*E*/*X*, or the energy surplus per unit time Δ*E*/*T*. These strategies will be derived in the next two sub-sections; the optimization problem is formalized herein.

The speed and depth schedules define the dive angle

8 $$\gamma (t)=-{\sin }^{-1}(\frac{1}{v(t)}\frac{{\rm{d}}h(t)}{{\rm{d}}t}),$$

the horizontal

9 $${v}_{x}(t)=v(t)\cos \,\gamma (t)$$

and vertical *v*_*z*_(*t*) = d*h*(*t*)/d*t* velocity components, and, of course, the longitudinal (caudo-cranial) d*v*/d*t* and normal (ventro-dorsal) *v*(*t*)d*γ*(*t*)/d*t* acceleration components. In order to keep these schedules, thrust

10 $$F(t)=D(t)+W\,\sin \,\gamma (t)+{m}_{x}{\rm{d}}v(t)/{\rm{d}}t$$

and lift

11 $$L(t)=W\,\cos \,\gamma (t)+{m}_{z}v(t){\rm{d}}\gamma (t)/{\rm{d}}t$$

have to balance hydrodynamic resistance (*D*), gravity (*W*sin*γ* and *W*cos*γ*), and acceleration (*m*_*x*_d*v*/d*t* and *m*_*z*_*v*d*γ*/d*t*). Here, *W* is the submerged weight of the fish, whereas *m*_*x*_ and *m*_*z*_ are the caudo-cranial and ventro-dorsal components of its apparent mass. The associated active (routine) metabolic rate is

12 $$P(t)={P}_{0}(\tau (t))+\frac{1}{\eta }(v(t)D(t)+Wv(t)\sin \,\gamma (t)+\frac{{m}_{x}}{2}\frac{{\rm{d}}{v}^{2}(t)}{{\rm{d}}t})$$

by (4) and (10). It will be assumed that the chemo-mechanical propulsion efficiency *η* = *η*_*h*_*η*_*c*_, which is a product of the hydrodynamic efficiency *η*_*h*_ and chemo-mechanical efficiency of the propulsive muscles *η*_*c*_, is a constant. This assumption can be accepted as plausible because carcharhinid sharks are almost anguilliform swimmers, and these swimmers may have practically condition-independent hydrodynamic propulsion efficiency^22^.

The caudo-cranial component of the apparent mass is only a few percent greater than the ‘real’ mass, *m*; the ventro-dorsal component of the apparent mass is approximately twice the real mass. The submerged weight of the fish can be expressed as

13 $$W=\frac{\beta }{1+\beta }mg\approx \beta mg,$$

where, *β* is the excess density of the shark (typically, a few hundredths), and *g* is the acceleration of gravity.

Drag and lift can always be expressed in terms of their coefficients, *C*_*D*_ and *C*_*L*_, with

14 $$D(t)=\frac{1}{2}\rho {v}^{2}(t)S{C}_{D}(t),$$

15 $$L(t)=\frac{1}{2}\rho {v}^{2}(t)S{C}_{L}(t),$$

in which *ρ* is the density of water and *S* is an arbitrary reference area. In turn,

16 $${C}_{D}(t)={C}_{D0}+K{C}_{L}^{2}(t),$$

where *C*_*D*0_ and *K* are the parasite (zero lift) and induced drag coefficients, which depend on morphology of the body and fins^12^, whereas

17 $${C}_{L}(t)=\frac{2W\,\cos \,\gamma (t)}{\rho S{v}^{2}(t)}+\frac{2{m}_{z}}{\rho Sv(t)}\frac{{\rm{d}}\gamma (t)}{{\rm{d}}t},$$

by (11). When swimming along a straight path, the last term in (17) falls out.

Introducing (10)–(17) in (12) and yields

18 $$\begin{array}{rcl}\langle P\rangle  & = & \langle {P}_{0}\rangle +\frac{\rho S{C}_{D0}}{2\eta }(\langle {v}^{3}\rangle +\frac{K}{{C}_{D0}}({(\frac{2W}{\rho S})}^{2}\langle \frac{{\cos }^{2}\gamma }{v}\rangle +{(\frac{2{m}_{z}g}{\rho S})}^{2}\langle \frac{v}{{g}^{2}}{(\frac{{\rm{d}}\gamma }{{\rm{d}}t})}^{2}\rangle ))\\  &  & +\frac{1}{\eta }(-W\frac{h(T)-h(0)}{T}+{m}_{x}\frac{{v}^{2}(T)-{v}^{2}(0)}{2T}+{m}_{z}W\frac{4K}{\rho S}\frac{\sin \,\gamma (T)-\,\sin \,\gamma (0)}{T})\end{array}$$

for the average active (routine) metabolic rate. After a sufficiently long swimming interval (formally, when *T *→ ∞), the last three terms fall out. Exploiting (9), the remaining terms can be recast as:

19 $$\langle P\rangle =\langle {P}_{0}\rangle (1+\frac{1}{2{w}^{3}}\langle \frac{{v}_{x}^{3}}{{\cos }^{{\rm{3}}}\gamma }\rangle +\frac{{u}^{4}}{2{w}^{3}}\langle \frac{{\cos }^{{\rm{3}}}\gamma }{{v}_{x}}\rangle +\frac{{u}^{4}}{2{w}^{3}{a}^{2}}\langle \frac{{v}_{x}}{\cos \,\gamma }{(\frac{{\rm{d}}\gamma }{{\rm{d}}t})}^{2}\rangle ),$$

where

20 $$w={(\frac{\eta \langle {P}_{0}\rangle }{\rho S{C}_{D0}})}^{1/3},$$

21 $$u={(\frac{K}{{C}_{D0}})}^{1/4}{(\frac{2W}{\rho S})}^{1/2},$$

22 $$a=W/{m}_{z}\approx \beta g/2.$$

The first two quantities have the dimensions of speed, but they defer a simple interpretation^12^. The last quantity has the dimension of acceleration, and it can be interpreted as an initial vertical acceleration of the submerged (motionless) shark under the action of gravity. The final form of (19),

23 $$\langle P\rangle =\langle {P}_{0}\rangle (1+\frac{{\langle {v}_{x}\rangle }^{3}}{2{w}^{3}}\langle \frac{{(1+\varpi )}^{3}}{{\cos }^{3}\gamma }\rangle +\frac{{u}^{4}}{2{w}^{3}\langle {v}_{x}\rangle }\langle \frac{{\cos }^{3}\gamma }{1+\varpi }\rangle +\frac{{u}^{4}\langle {v}_{x}\rangle }{2{w}^{3}{a}^{2}}\langle \frac{1+\varpi }{\cos \,\gamma }{(\frac{{\rm{d}}\gamma }{{\rm{d}}t})}^{2}\rangle ),$$

follows by substitution

24 $${v}_{x}(t)=\langle {v}_{x}\rangle (1+\varpi (t)),$$

where *ϖ* can be interpreted as the dimensionless variation of the (reduced) horizontal speed. Note that

25 $$\langle \varpi \rangle =0$$

by definition.

As mentioned already, we seek two canonical swimming strategies that maximize either the energy surplus per unit distance Δ*E*/*X* or the energy surplus per unit time Δ*E*/*T*. Both quantities involve the specific energy densities *e*_*X*_ and *e*_*T*_, which will be assumed known. *e*_*T*_ enters Δ*E* only in combination 〈*P*〉 − *e*_*T*_ – see (6) – its presence is equivalent to a reduction in the basic metabolic rate. By replacing 〈*P*_0_〉 in (18) and (20) with 〈*P′*_0_〉 = 〈*P*_0_〉 − *e*_*T*_ (and marking the associated *P* and *w* by primes), *e*_*T*_ is effectively eliminated from the formulation and the two optimization problems respectively reduce to those of maximizing Δ*E*/*X* = *e*_*X*_ − *C′* and Δ*E*/*T* = (*e*_*X*_ − *C′*)〈*v*_*x*_〉 in which

26 $$C^{\prime} =\langle P^{\prime} \rangle /\langle {v}_{x}\rangle $$

is the (modified) cost of transport (compare (7)).

#### Maximizing the energy surplus per unit distance

Given *e*_*X*_ and *e*_*T*_ < 〈*P*_0_〉, the swimming strategy – now manifested in *ϖ*(·) and *γ*(·) schedules and in the average horizontal velocity component 〈*v*_*x*_〉 – that maximizes Δ*E*/*X* will be the strategy that minimizes the modified cost of transport, *C*′. This problem is similar to that addressed in^21^ and it will be solved using a similar approach. The main (and major) difference between the two is in the modelling of the drag coefficient (it was assumed constant in^21^ but is allowed to change with speed herein), and in the average swimming speed becoming an inseparable part of the swimming strategy (the average swimming speed was not addressed in^21^).

We begin with an observation that, except for a few singular events of limited duration, the speed of the four tagged sharks was remarkably constant and their dive angles were small and changed slowly – see Figs 1–3. Consequently, we assume, subject to an a posteriori verification, that these features also characterize the optimal swimming strategy we are looking for. In other words, we assume that |*ϖ*| and *γ* are small as compared with unity, and that the characteristic time scale *t*_*γ*_ on which *γ* changes exceeds 〈*v*_*x*_〉/*a* ≈ 2〈*v*_*x*_〉/*gβ* (see (22)), which is a few seconds (typical values of 〈*v*_*x*_〉 can be found in Table 2, typical values of *β* can be found in Supplementary Material Table 3). Under these assumptions,

27 $$\begin{array}{rcl}C^{\prime}  & = & \langle {P}_{0}\rangle (\frac{1}{\langle {v}_{x}\rangle }+\frac{{\langle {v}_{x}\rangle }^{2}}{2{w^{\prime} }^{3}}(1+3\langle {\varpi }^{2}\rangle +\frac{3}{2}\langle {\gamma }^{2}\rangle )\\  &  & +\,\frac{{u}^{4}}{2{w^{\prime} }^{3}{\langle {v}_{x}\rangle }^{2}}(1+\langle {\varpi }^{2}\rangle -\frac{3}{2}\langle {\gamma }^{2}\rangle )+\frac{{u}^{4}}{2{w^{\prime} }^{3}{a}^{2}}\langle {(\frac{{\rm{d}}\gamma }{{\rm{d}}t})}^{2}\rangle +\ldots ),\end{array}$$

where the ellipsis stands for the higher order averages of *ϖ* and *γ*(and d*γ*/d*t*). In deriving (27) we have exploited (25).

**Table 2:** Swimming statistics for the four oceanic whitetip sharks.

Shark	Duration (s)	〈*v*〉 (m/s)	〈*v*_*x*_〉 (m/s)	〈*ϖ*^2^〉	〈*γ*^2^〉	〈(d*γ*/d*t*)^2^〉 (1/s^2^)
OWT1	218428	0.66	0.66	0.035	0.014	5 10^−4^
OWT2	43932	0.74	0.73	0.010	0.021	5 10^−4^
OWT3	134977	0.76	0.75	0.029	0.014	2 10^−4^
OWT4	118001	0.63	0.63	0.010	0.005	9 10^−5^

Being based on the second derivative of depth, the values of 〈(d*γ*/d*t*)^2^〉 are sensitive to the number of points used in estimation of the derivative. The values found in the table are based on differentiating a running 11-point parabolic fit of the depth. Doubling the number of points reduces 〈(d*γ*/d*t*)^2^〉 eight-fold. Typical values of 〈*v*_*x*_〉^2^/*a*^2^ that multiply 〈(d*γ*/d*t*)^2^〉 in (27) are bounded between 8 and 32, depending on the sinking factor.

The optimal average horizontal speed component 〈*v*_*x*_〉 will be the one at which ∂*C′*/〈*v*_*x*_〉 = 0. In other words, it will be the solution of

28 $$\langle {v}_{x}\rangle {w{\rm{^{\prime} }}}^{3}-{\langle {v}_{x}\rangle }^{4}(1+3\langle {\varpi }^{2}\rangle +\frac{3}{2}\langle {\gamma }^{2}\rangle +\ldots )+{u}^{4}(1+\langle {\varpi }^{2}\rangle -\frac{3}{2}\langle {\gamma }^{2}\rangle +\ldots )=0,$$

where the ellipsis stands for the higher order terms with respect to *ϖ* and *γ*. The leading-order solution of this equation is

29 $${\langle {v}_{x}\rangle }_{{\rm{\min }}C^{\prime} }={v^{\prime} }_{\ast }(1-\frac{3{v^{\prime} }_{\ast }^{4}-{u}^{4}}{4{v^{\prime} }_{\ast }^{4}-{v^{\prime} }_{\ast }{w^{\prime} }^{3}}\langle {\varpi }^{2}\rangle -\frac{3}{2}\frac{{v^{\prime} }_{\ast }^{4}+{u}^{4}}{4{v^{\prime} }_{\ast }^{4}-{v^{\prime} }_{\ast }{w^{\prime} }^{3}}\langle {\gamma }^{2}\rangle +\ldots ),$$

where v'_*_ satisfies

30 $${w^{\prime} }^{3}{v^{\prime} }_{\ast }-{v^{\prime} }_{\ast }^{4}+{u}^{4}=0.$$

By interpretation, *v*′_*_ is the speed that minimizes the (modified) cost of transport when swimming at constant depth^12^. It can be closely approximated by

31 $${v^{\prime} }_{\ast }\approx w^{\prime} (1+\frac{3}{14}\frac{{u}^{3}}{{w^{\prime} }^{3}})$$

(see Table 1 in^12^) for all practical combinations of *u* and *w*′, but the series

32 $${v^{\prime} }_{\ast }=w^{\prime} (1+\frac{1}{3}\frac{{u}^{4}}{{w^{\prime} }^{4}}+O(\frac{{u}^{8}}{{w^{\prime} }^{8}}))$$

applicable when ${u}^{4}\ll {w^{\prime} }^{4}$ (ibid.), will be more useful for qualitative analysis.

The associated average swimming speed

33 $$\begin{array}{ccc}{\langle v\rangle }_{minC^{\prime} } & = & {\langle {v}_{x}\rangle }_{minC^{\prime} }\langle \frac{1+\varpi }{\cos \,\gamma }\rangle ={\langle {v}_{x}\rangle }_{minC^{\prime} }(1+\frac{1}{2}\langle {\gamma }^{2}\rangle +\ldots )\\  & = & {v^{\prime} }_{\ast }(1-\frac{(3{v^{\prime} }_{\ast }^{4}-{u}^{4})\langle {\varpi }^{2}\rangle +{u}^{4}\langle {\gamma }^{2}\rangle }{4{v^{\prime} }_{\ast }^{4}-{v^{\prime} }_{\ast }{w^{\prime} }^{3}}+\ldots )\end{array}$$

follows (29) by (9), (25) and (30). Again, the ellipsis stands for the higher order terms with respect to *ϖ* and *γ*. When ${u}^{4}\ll {w^{\prime} }^{4}$, it becomes

34 $${\langle v\rangle }_{{\rm{\min }}C^{\prime} }={v^{\prime} }_{\ast }(1-\langle {\varpi }^{2}\rangle +\ldots )$$

by (32); terms of the order 〈*ϖ*^2^〉(*u*^4^/*w*′^4^) and 〈*γ*^2^〉(*u*^4^/*w*′^4^) become masked behind the ellipsis.

The associated (modified) cost of transport

35 $$\mathop{{\rm{\min }}}\limits_{\langle {v}_{x}\rangle }C^{\prime} ={C^{\prime} }_{\ast }(1+\frac{(3{v^{\prime} }_{\ast }^{4}+{u}^{4})\langle {\varpi }^{2}\rangle +\frac{3}{2}\langle {\gamma }^{2}\rangle ({v^{\prime} }_{\ast }^{4}-{u}^{4})+\frac{{u}^{4}{v^{\prime} }_{\ast }^{2}}{{a}^{2}}\langle {({\rm{d}}\gamma /{\rm{d}}t)}^{2}\rangle }{2{w^{\prime} }^{3}{v^{\prime} }_{\ast }+{v^{\prime} }_{\ast }^{4}+{u}^{4}}+\ldots )$$

follows (27) and (29). Here,

36 $${C^{\prime} }_{\ast }=\frac{\langle {P^{\prime} }_{0}\rangle }{{v^{\prime} }_{\ast }}(1+\frac{{v^{\prime} }_{\ast }^{3}}{2{w^{\prime} }^{3}}+\frac{{u}^{4}}{2{v^{\prime} }_{\ast }{w^{\prime} }^{3}})$$

is the minimal cost of transport when swimming at constant depth and speed^12^. It can be closely approximated by

37 $${C^{\prime} }_{\ast }\approx \frac{3}{2}\frac{\langle {P^{\prime} }_{0}\rangle }{{v^{\prime} }_{\ast }}(1+\frac{8}{15}\frac{{u}^{7/2}}{{w^{\prime} }^{7/2}})$$

(the last row in the 6^th^ column of Table 1 in^12^). Nonetheless, a series expansion

38 $${C^{\prime} }_{\ast }=\frac{3}{2}\frac{\langle {P^{\prime} }_{0}\rangle }{{v^{\prime} }_{\ast }}\,(1+\frac{2}{3}\frac{{u}^{4}}{{w^{\prime} }^{4}}+O(\frac{{u}^{8}}{{w^{\prime} }^{8}})),$$

applicable when ${u}^{4}\ll {w^{\prime} }^{4}$, will be more useful for a qualitative analysis. In this case, (35) becomes

39 $$\mathop{{\rm{\min }}}\limits_{\langle {v}_{x}\rangle }C^{\prime} ={C^{\prime} }_{\ast }\,(1+\langle {\varpi }^{2}\rangle +\frac{\langle {\gamma }^{2}\rangle }{2}+\ldots );$$

by (32); the term involving 〈(d*γ*/d*t*)^2^〉 turns to be of a higher order and hence negligible.

Noting that *v*′_*_ exceeds both *u* and *w*′, equation (35) implies that variations in speed, dive angle and its rate of change increase the cost of transport. It recapitulates the conclusion of^21^ that the swimming strategy that minimizes the cost of transport is swimming at constant speed and depth. Because no pelagic predator swims in this way, one must conclude that yo-yo diving (manifested in 〈*γ*^2^〉 > 0 and 〈(d*γ*/d*t*)^2^〉 > 0) increases the probability of capturing prey, *e*_*X*_. When yo-yo diving, the best strategy would still be swimming at constant speed (〈*ϖ*^2^〉 → 0), but slightly slower than would have been needed to minimize the cost of transport at constant depth (33).

The minimal (modified) cost of transport ${{\rm{\min }}}_{\langle {v}_{x}\rangle }C^{\prime}$ is practically the minimal cost of transport when swimming at constant depth, *C*′_*_. It diminishes with the (modified) average basic metabolic rate, 〈*P*′_0_〉 = 〈*P*_0_〉 − *e*_*T*_ (this conjecture has been formally demonstrated in^12^; it can be verified by setting *u*^4^ → 0 in (38)). Consequently, the swimming strategy that minimizes the cost of transport of an ectothermic predator would be swimming as deep (as cold) as possible. Because no pelagic shark swims in this way as well, swimming at shallower depth either increases the probabilities *e*_*X*_ and *e*_*T*_, or accelerates the digestion rate.

The optimal swimming speed ${\langle v\rangle }_{{\rm{\min }}C^{\prime} }$ is practically the swimming speed that would have minimized the modified cost of stransport when swimming at constant depth. This speed decreases with the (modified) average basic metabolic rate (manifested here in *w*′, which is related with 〈*P*′_0_〉 by (20)) and hence decreases with decreasing temperature (increasing depth) and with increasing *e*_*T*_. In other words, the optimal swimming speed of a slow moving predator (for which *e*_*T*_ is not negligible) will be slower than the speed that minimizes its (unmodified) cost of transport.

#### Maximizing the energy surplus per unit time

Here, we seek the swimming strategy that maximizes Δ*E*/*T* = (*e*_*X*_〈*v*_*x*_〉 − (〈*P*〉 − *e*_*T*_)) = 〈*v*_*x*_〉(*e*_*X*_ − *C*′) under the assumption that *e*_*X*_ and *e*_*T*_ are given. The minimal energy density *e*_*X*_ that may keep Δ*E* nonnegative is the minimal cost of transport found in (35), $\mathop{{\rm{\min }}}\limits_{\langle {v}_{x}\rangle }C^{\prime}$. Swimming at a speed that differs from the optimal swimming speed in (33), ${\langle {v}_{x}\rangle }_{{\rm{\min }}C}$ will make the surplus negative. Hence, the optimal strategy that maximizes Δ*E*/*T* when ${e}_{X}=\mathop{{\rm{\min }}}\limits_{\langle {v}_{x}\rangle }C^{\prime}$ is also the strategy that maximizes Δ*E*/*X*; in both cases, Δ*E* = 0.

When *e*_*X*_ exceeds $\mathop{{\rm{\min }}}\limits_{\langle {v}_{x}\rangle }C^{\prime}$, the (horizontal) swimming velocity that maximizes Δ*E*/*T* is the one for which the derivative of Δ*E*/*T* with respect to 〈*v*_*x*_〉 vanishes; i.e.

40 $$({e}_{X}-C^{\prime} )-\langle {v}_{x}\rangle \frac{\partial C^{\prime} }{\partial \langle {v}_{x}\rangle }=0.$$

Here, *e*_*X*_ − *C*′ is positive by assumption, and hence this equation can be satisfied only when ∂*C*′/∂〈*v*_*x*_〉 > 0. But ∂*C*′/∂〈*v*_*x*_〉 = 0 at $\langle {v}_{x}\rangle ={\langle {v}_{x}\rangle }_{{\rm{\min }}C^{\prime} }$ by definition, and therefore ∂*C*′/∂〈*v*_*x*_〉 will be positive only at $\langle {v}_{x}\rangle  > {\langle {v}_{x}\rangle }_{{\rm{\min }}C^{\prime} }$. In other words, provided a sufficient prey density, the swimming speed that maximizes the energy surplus rate ${\langle {v}_{x}\rangle }_{{\rm{\max }}({\rm{\Delta }}E/T)}$ will be higher than the speed that minimizes the (modified) cost of transport ${\langle {v}_{x}\rangle }_{{\rm{\min }}C^{\prime} }$.

Assuming that ${\langle {v}_{x}\rangle }_{{\rm{\max }}({\rm{\Delta }}E/T)}-{\langle {v}_{x}\rangle }_{{\rm{\min }}C^{\prime} }$ is sufficiently small, equation (40) can be solved asymptotically. To this end, we expand the (modified) cost of transport into a power series about ${\langle {v}_{x}\rangle }_{{\rm{\min }}C^{\prime} }$

41 $$C^{\prime} =\mathop{{\rm{\min }}}\limits_{\langle {v}_{x}\rangle }C^{\prime} +\frac{1}{2}{(\frac{{\partial }^{2}C^{\prime} }{\partial {\langle {v}_{x}\rangle }^{2}})}_{\langle {v}_{x}\rangle ={\langle {v}_{x}\rangle }_{{\rm{\min }}C^{\prime} }}{(\langle {v}_{x}\rangle -{\langle {v}_{x}\rangle }_{{\rm{\min }}C^{\prime} })}^{2}+\ldots ;$$

the linear term falls out because ∂*C*′/∂〈*v*_*x*_〉 = 0 at $\langle {v}_{x}\rangle ={\langle {v}_{x}\rangle }_{{\rm{\min }}C^{\prime} }$. Introducing (41) in (40) yields, in the leading order with respect to the energy surplus

42 $${\langle {v}_{x}\rangle }_{{\rm{\max }}({\rm{\Delta }}E/T)}-{\langle {v}_{x}\rangle }_{{\rm{\min }}C^{\prime} }=\frac{{e}_{X}-\mathop{{\rm{\min }}}\limits_{\langle {v}_{x}\rangle }C^{\prime} }{{\langle {v}_{x}\rangle }_{{\rm{\min }}C^{\prime} }{(\frac{{\partial }^{2}C}{\partial {\langle {v}_{x}\rangle }^{2}})}_{\langle {v}_{x}\rangle ={\langle {v}_{x}\rangle }_{{\rm{\min }}C^{\prime} }}}+\ldots .$$

In other words, the difference in the swimming speed between the two strategies increases proportionally to the energy surplus (per distance), ${e}_{X}-\mathop{{\rm{\min }}}\limits_{\langle {v}_{x}\rangle }C^{\prime}$.

### Data accessibility

The datasets generated during and/or analyzed during the current study are available from the corresponding author on reasonable request.

## Electronic supplementary material

Supplementary material

Shark borne video footage

## References

[1] Pyke GH (1981). Optimal travel speeds of animals. American Naturalist.

[2] Dickson KA (1995). Unique adaptations of the metabolic biochemistry of tunas and billfishes for life in the pelagic environment. Environmental Biology of Fishes.

[3] Brill RW (1996). Selective advantages conferred by the high performance physiology of tunas, billfishes and dolphin fish. Comparative Biochemistry and Physiology A.

[4] Wegner NC, Sepulveda CA, Bull KB, Graham JB (2010). Gill morphometrics in relation to gas transfer and ram ventilation in high-energy demand teleosts: scombrids and billfishes. Journal of Morphology.

[5] Whitlock RE (2015). Direct quantification of energy intake in an apex marine predator suggests physiology is a key driver of migrations. Science Advances.

[6] Dowd WW, Brill RW, Bushnell PG, Musick JA (2006). Standard and routine metabolic rates of juvenile sandbar sharks (*Carcharhinus plumbeus*) including the effects of body mass and acute temperature change. Fisheries Bulletin.

[7] Lessa R, Santana FM, Paglerani R (1999). Age, growth and stock structure of the oceanic whitetip shark, C*archarhinus longimanus*, from the southwestern equatorial Atlantic. Fisheries Research.

[8] Howey-Jordan LA (2013). Complex movements, philopatry and expanded depth range of a severely threatened pelagic shark, the oceanic whitetip (*Carcharhinus longimanus*) in the western north Atlantic. PLoS One.

[9] Gleiss AC, Norman B, Wilson RP (2011). Moved by that sinking feeling: variable diving geometry underlies movement strategies in whale sharks. Functional Ecology.

[10] Nakamura I, Watanabe YY, Papastamatiou YP, Sato K, Meyer CG (2011). Yo-yo vertical movements suggest a foraging strategy for tiger sharks *Galeocerdo cuvier*. Marine Ecology Progress Series.

[11] Carey FG, Scharold JV (1990). Movements of blue sharks (*Prionace glauca*) in depth and course. Marine Biology.

[12] Iosilevskii G, Papastamatiou YP (2016). Relations between morphology, buoyancy and energetics of requiem sharks. Royal Society Open Science.

[13] Madigan DJ (2015). Diet shift and site-fidelity of oceanic whitetip sharks, *Carcharhinus longimanus* along the Great Bahamas Bank. Marine Ecology Progress Series.

[14] Williams TM (2014). Instantaneous energetics of puma kills reveal advantage of felid sneak attacks. Science.

[15] Towner AV (2016). Sex-specific and individual preferences for hunting strategies in white sharks. Functional Ecology.

[16] Blank JM, Farwell CJ, Morrissette JM, Schallert RJ, Block BA (2007). Influence of swimming speed on metabolic rates of juvenile Pacific Bluefin tuna and yellowfin tuna. Physiological Biochemical Zoology.

[17] Watanabe YY, Goldman KJ, Caselle JE, Chapman DD, Papastamatiou YP (2015). Comparative analyses of animal-tracking data reveal ecological significance of endothermy in fishes. Proceedings of the National Academy of Sciences.

[18] Olson RJ, Boggs CH (1986). Apex predation by yellowfin tuna (*Thunnus albacares*): independent estimates from gastric evacuation and stomach contents, bioenergetics, and cesium concentrations. . Canadian Journal Fisheries and Aquatic Sciences.

[19] Seki T, Taniuchi T, Nakano H, Shimizu M (1998). Age, growth and reproduction of the oceanic whitetip shark from the Pacific Ocean. Fisheries Science.

[20] Watanabe YY (2008). Swimming behavior in relation to buoyancy in an open swimbladder fish, the Chinese sturgeon. Journal of Zoology.

[21] Iosilevskii G, Papastamatiou YP, Meyer CG, Holland KN (2012). Energetics of the yo-yo dives of predatory sharks. Journal Theoretical Biology.

[22] Iosilevskii G (2016). Hydrodynamics of a flexible soft-rayed caudal fin. PLoS one.

