# Supplementary material for: Optimal swimming strategies and behavioral plasticity of oceanic whitetip sharks
Source: Sci Rep. 2018 Jan 11;8:551. doi: 10.1038/s41598-017-18608-z (PMC5765167; doi:10.1038/s41598-017-18608-z)
Supplement: Supplementary file 1 — Supplementary material [file 41598_2017_18608_MOESM1_ESM.pdf]

Supplementary material for

Optimal swimming strategy and behavioral plasticity of oceanic whitetip sharks

Yannis P. Papastamatiou[1], Gil Iosilevskii[2], Vianey Leos-Barajas[3], Edd J. Brooks[4], Lucy A. Howey[5], Demian D. Chapman[1], Yuuki Y. Watanabe[6,7]

[1] Department of Biological Science, Florida International University, Florida, USA
[2] Faculty of Aerospace Engineering, Technion, Haifa, Israel
[3] Department of Statistics, Iowa State University, Iowa, USA
[4] Shark Research and Conservation Program, Cape Eleuthera Institute, Eleuthera, the Bahamas
[5] Microwave Telemetry Inc., Maryland, USA
[6] National Institute of Polar Research, Tachikawa, Tokyo, Japan
[7] Department of Polar Science, SOKENDAI (The Graduate University for Advanced Studies), Tachikawa, Tokyo, Japan

Supplementary figures

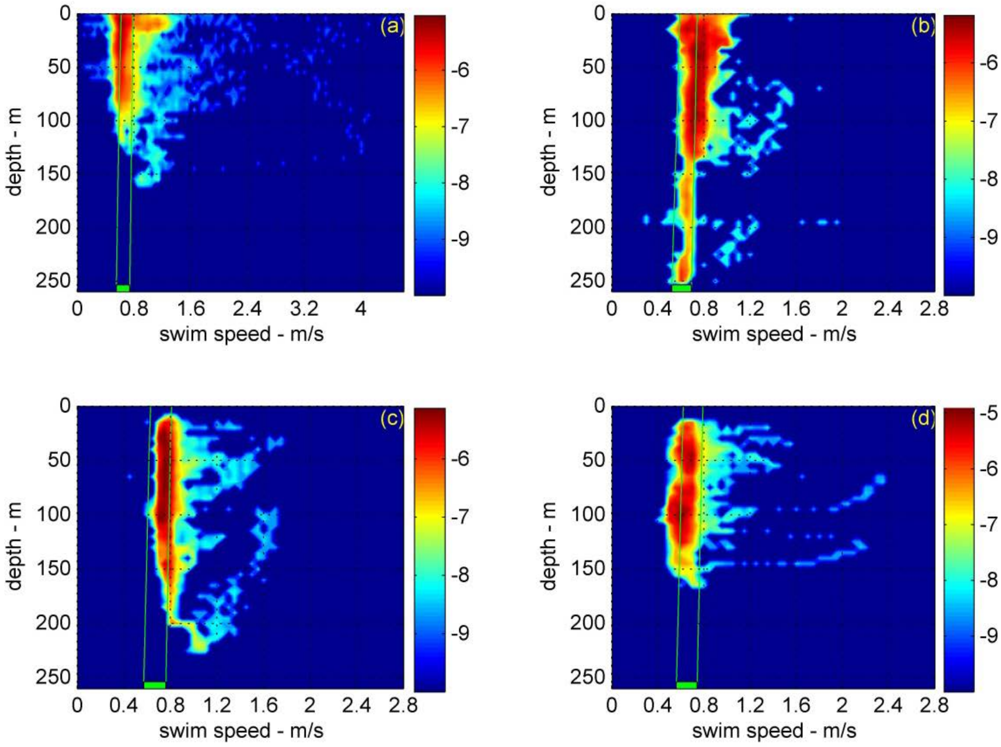

Supplementary figure S1: Density distribution of shark swim speeds with depth (panels (a)-(d) correspond to sharks OWT1-4). This is the same as Fig. 2 in the main text, only the speed range is different and the color marks the (base 10) logarithm of the density. Bursts of swim speed (likely foraging) occur at all depths.

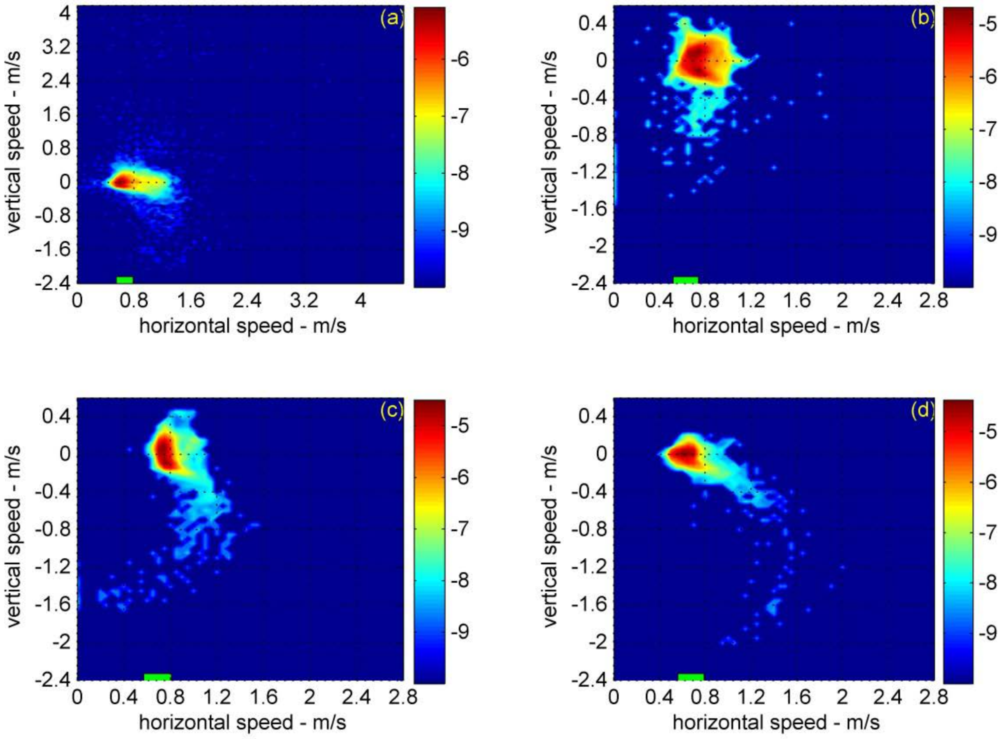

Supplementary figure S2: Density distribution of shark's velocity (panels (a)-(d) correspond to sharks OWT1-4). It is similar to Fig. 3 in the main text, only the axes are somewhat different and the color marks the (base 10) logarithm of the density. Bursts of swim speed are downwards for sharks OWT2-4 (plates b-d), but also upwards for shark OWT1 (plate a).

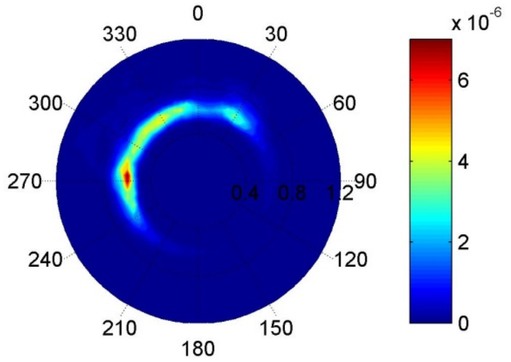

Supplementary figure S3: Density distribution of heading and speed for OWT1. In general, the shark swam North West at 0.6 m/s.

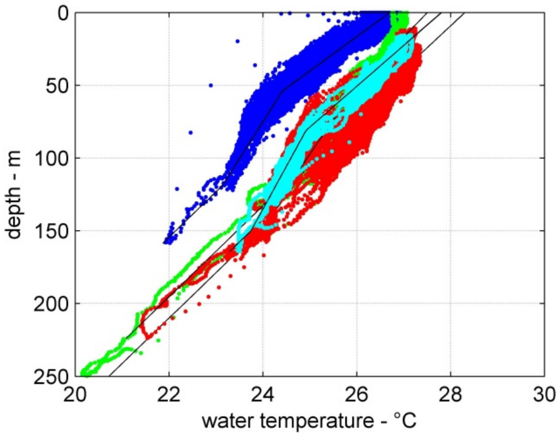

Supplementary figure S4: Water temperature as a function of depth encountered by the four sharks (down-sampled to 0.1 HZ). Blue, green, red and cyan stand for sharks OWT1-4, respectively. A few separate points corresponding to OWT1 mark its fast ascents from 160 and 90 meters, respectively. They manifest the time lag (about 30 seconds) of the temperature sensor. Black solid lines mark the three-segment linear regression of (S1).

Supplementary tables

Supplementary table S1: Morphometrics of 6 sharks caught at Cat Island. *l*, *s*, *b*, and c_0 are the pre-caudal length, length, span and proximal chord of the pectoral fins.

l (cm)	s/l	b/l	c_0/l
205	0.259	0.683	0.132
190	0.237	0.632	0.126
202	0.233	0.673	0.134
177	0.254	0.621	0.136
203	0.246	0.631	0.123
167	0.240	0.623	0.126
average	0.245	0.644	0.129
range	+0.014 -0.012	+0.039 -0.022	±0.006

Supplementary table S2: Fins dimensions used in the estimation. *s* is the length of a fin – for the caudal fin it is the dorso-ventral distance between the upper and the lower lobes, for all other fins it is the distance between the distal margin of the fin and the body; c_0 is the proximal chord of the fin; *l* is the pre-caudal length; t/c is the thickness to chord ratio.

parameter	caudal	pectoral	dorsal 1	dorsal 2	anal	pelvic
s/l	0.24	0.22 to 0.28	0.13	0.04	0.04	0.06
c_0/l	0.13	0.12 to 0.14	0.13	0.08	0.06	0.06
t/c	0.2	0.1	0.1	0.1	0.1	0.1

Supplementary table S3: Estimated data; 'min' and 'max' mark the minimal and maximal bracketed values. β, b, d and S are the sinking factor, span of the pectoral fins, body diameter and maximal cross section area of the body, respectively. SC_{D0} and K are the drag area and the induced drag coefficient. P_0 is the standard metabolic rate in mmols ATP per second. Formation of 1 mmol ATP requires, approximately, 5 milligrams O_2 and 16 calories of a substrate (fats, proteins or carbohydrates).

	OWT1		OWT2		OWT3		OWT4		
parameter	min	max	min	max	min	max	min	max	comments
mass (kg)	98	120	54	66	113	138	89	109	(S2)
β	0.025	0.05	0.025	0.05	0.025	0.05	0.025	0.05	guessed
b (m)	1.15	1.37	0.91	1.08	1.22	1.45	1.11	1.33	(S5)
d (m)	0.33	0.37	0.28	0.31	0.35	0.38	0.32	0.36	(S4)
S (m^2)	0.09	0.11	0.063	0.077	0.094	0.115	0.082	0.1	$\pi d^2/4$
SC_{D0} (m^2)	0.019	0.030	0.014	0.02	0.020	0.032	0.018	0.028	(S7), (S13)
K	0.023	0.036	0.027	0/041	0.022	0.035	0.023	0.036	(S6)
P_0 at 26 °C	0.25	0.30	0.16	0.19	0.29	0.36	0.24	0.28	(S3)

Supplementary notes: Estimated parameters

Properties of water

Water density ρ was assumed constant, 1025 kg/m^3, whereas its viscosity was approximated with 0.044/(T-249) kg/m/s, where T is water temperature in degrees Kelvin. This equation represents a curve-fit of the data found on page 587 of [1].

Water temperature

Water temperature was measured directly and used instead of the body temperature. This substitution is in error during rapid ascents or descents, but during these maneuvers, the basic metabolic rate (which is the primary parameter affected by the body temperature) comprises only a tiny fraction of the active metabolic rate, rendering the temperature error inconsequential. To draw the range boundaries in figure 2 of the main text, the temperature-depth relation was approximated by

$$T(h) = \begin{cases} T_0 - T_1 h & h \in [0, h_1), \\ T_0 - T_1 h_1 - T_2 (h - h_1) & h \in [h_1, h_2), \\ T_0 - T_1 h_1 - T_2 (h_2 - h_1) - T_3 (h - h_2) & h \geq h_2, \end{cases} \tag{S1}$$

where the coefficients h_1, h_2, T_0, T_1, T_2 have been curve-fitted for each shark (see supplementary figure S4). The numbers are (54, 120, 26.7, 0.042, 0.02, 0.032) for OWT1; (90, 147, 28.3, 0.033, 0.023, 0.032) for OWT2; (50, 110, 27.5, 0.03, 0.021, 0.032) for OWT3; and (81, 150, 27.8, 0.036, 0.017, 0.032) for OWT4.

Buoyancy

The sinking factor β of the oceanic whitetip sharks was estimated based on densities of other carcharhinids (as reported in [2]). Specifically, it was assumed to be between 0.025 (blue sharks, *Prionace glauca*) and 0.05 (dusky or bull sharks, *Carcharhinus obscurus, C. leucus*).

Mass

The mass of the sharks was estimated to be between 0.9 and 1.1 of the standard mass based on the regression

$$m = al^b \,, \tag{S2}$$

where l is the pre-caudal length (approximately 0.714 of the total length), whereas $a = 3.077 \cdot 10^{-5}$ and $b = 2.86$ are the respective coefficients [3].

Standard metabolic rate

Standard metabolic rate was estimated with

$$P_0 = k_P m^{\alpha} e^{-k_\tau/\tau} \tag{S3}$$

where τ is the absolute body temperature, whereas k_P = 127 mol ATP per second$\cdot$ kg$^{\alpha}$, α = 0.8, and k_τ = 5020 °K were chosen after [4]. We have used a regression based on many different teleost species and a large range of body masses (they can be interpolated for the mass of the oceanic white tip sharks, about 100 kg) over a regression based on juvenile carcharhinid sharks [5] in the 1 to 10 kg range. In any case, the values of the basic metabolic rate should be taken with caution, because there can be interspecific differences in these parameters ([4], [6]).

Chemo-mechanical efficiency

Chemo-mechanical efficiency of the muscles (the mechanical work done per mole ATP) η_c was assumed 24 Joules per mmol ATP independent of swimming conditions [7].

Metabolic efficiency

ATP is produced from various substrates in a series of irreversible reactions. It was assumed that production of one mmol of ATP requires approximately 70 Joules (16 calories) from proteins, fats or carbohydrates [8].

Morphometrics

Effective body diameter

Given m, β, and l, the diameter was estimated with

$$d = \left(\frac{4}{\pi} \frac{m}{\rho l k_m (1+\beta)} \right)^{1/2}, \qquad \text{(S4)}$$

where the prismatic coefficient k_m was chosen as that of a double ogive, 8/15.

Pectoral fins

Pectoral fins length s and chord c_0 were measured from 6 additional oceanic whitetip sharks we caught at Cat Island. The data is listed in supplementary table S1. We used the average values, bracketed ± 10% - approximately twice the measured range. Thus, the length of the fins was assumed to be between 22 and 27 % pre-caudal length (PCL), and the chord was assumed to be between 11.6 and 14.2 % PCL. To remain consistent, the span of the pectoral fins b was estimated from the body diameter and the length of the fin (the distance between the fin's tip and the body) with

$$b = 1.9s + d\ ; \qquad \text{(S5)}$$

it yielded practically the same values as those estimated based on the measured span.

Other fins

Dorsal, anal, pelvic and caudal fins dimensions are specified in supplementary table S2. The dimensions are approximate and possible inaccuracies have been accounted for by bracketing the drag coefficient (see below).

Propulsion efficiency and drag

Propulsion efficiency

Being both defined as the components of hydrodynamic force in the direction of swimming, thrust and drag of an actively swimming shark are essentially inseparable [9]. Remaining

consistent with [10], we define drag as the respective component of the hydrodynamic force that would have acted on the shark if it were gliding stretched at the same speed and the same body angle (see footnote 1 ibid.). Concurrently, we define thrust as the difference between the respective component of the hydrodynamic force acting on the (actively swimming) shark and the straight-body drag. In this way, any possible variations in friction between the body and the water are accounted for by the propulsion efficiency. When swimming at high Reynolds numbers, these variations are expected to be small [9], and the propulsion efficiency η_h is expected to be practically the same as the ideal efficiency [11]. We have assumed η_h a constant 0.70 for all individuals [12]. Propulsion efficiency was not bracketed, because it enters the relevant equations in combination with the parasite drag coefficient (see (20) in the main text), which has been bracketed between 1 and 1.25 of the nominal value estimated below.

Induced drag

The induced drag coefficient in equation (16) of the main text, *K*, was estimated based on pectoral fins dimensions with

$$K = \frac{k_K}{\pi} \frac{S}{b^2}, \qquad \text{(S6)}$$

where *b* is given by (S5), *S* is the reference area (we have used $S = \pi d^2 / 4$, the cross section area of the body), and k_K is a numerical factor accounting for increased flow separation from the surface of the fin due to angle of attack, for non-elliptical distribution of lift along the span, and, to some extent, for fins interaction. Based on unpublished experimental data, we have used $k_K = 1.5$ – see the supplementary material to [10].

Parasite drag

The parasite drag coefficient in equation (16) of the main text, C_{D0}, was estimated based on

the preliminary design tools of aircraft design [13], exactly as it was done in [10]. A short recapitulation follows.

C_{D0} is contributed by the body of the shark (it will be marked by the index '0'), its 8 fins (they will be marked by the indices '1',…,'8'), and the data-logger, which will be accounted for separately. Based on equations (12.24) and (12.27) of [13],

$$C_{D0} = \sum_{n=0}^{8} \frac{S_n}{S} C_f(\mathrm{Re}_n) F_n I_{n0} \,, \tag{S7}$$

where S is the reference area; S_0, …, S_8 are the wet areas of the respective constituents (namely, the contact areas between the corresponding parts and the water); F_0, …, F_8 are the form factors (empirical corrections for flow separation) that will be specified below;

$$\mathrm{Re}_n = \rho v l_n / \mu \,, \tag{S8}$$

is the Reynolds number based on the stream-wise dimension of the respective constituent, l_n; $I_{00},\ldots,I_{80}$ are the interference factors (empirical corrections accounting for hydrodynamic interference between the body and the fins and for hydrodynamic resistance of the gills); and, finally,

$$C_f(\mathrm{Re}) \approx 0.455 \big/ (\log_{10} \mathrm{Re})^{2.58} \tag{S9}$$

is the effective friction coefficient. In (S8), μ is the viscosity of water. Equation (S9) is based on a tacit assumption that the boundary layer is turbulent. To account for possible inaccuracies, we have bracketed the parasite drag coefficient between the value specified in (S9) and the one that is 25 % higher.

Approximating the shape of the body by double-ogive of length $l_0 = l$ and maximal effective diameter $d_0 = d$,

$$S_0 = \frac{2}{3} \pi l_0 d_0 \,. \tag{S10}$$

The respective form factor is

$$F_0 = 1 + 60\left(\frac{d_0}{l_0}\right)^3 + \frac{1}{400}\left(\frac{l_0}{d_0}\right) \tag{S11}$$

by (12.31) of [13]. $I_{00} = 1.2$ was introduced to account for hydrodynamic resistance of the gills.

$S_1, \ldots, S_N$ are, approximately twice the projected areas, $0.6\,c_{n0}s_n$, of the respective fins (the two parameters in this equation are the chord and length of the *n*th fin, and the numerical factor comes to compensate for its non-triangular shape). The form factors are

$$F_n \approx 1 + 2\frac{t_n}{l_n} + 100\left(\frac{t_n}{l_n}\right)^3 \tag{S12}$$

by Eq. (12.30) of [13]; $t_1, \ldots, t_N$ are the thicknesses of the fins. $I_{n0} = 1.4$ was set for every n >0 based on the suggestion appearing on page 283 ibid.

Datalogger drag

The datalogger is not streamlined, and therefore its parasite drag coefficient was estimated as unity when based on its frontal area, S_l =0.0038 m^2. In other words, S_l is also the increase in the drag area of the shark,

$$\Delta\left(SC_{D0}\right) = S_l\,. \tag{S13}$$

It accounts for more than 15% of the parasite drag of a 3 m shark.

References

[1] Batchelor G.K., *An introduction to fluid dynamics*, Cambridge University Press. p 597 (1990).

[2] Baldridge D.H., Sinking factors and average densities of Florida sharks as functions of liver buoyancy. *Copeia* **4**, pp. 744-754 (1970).

[3] Seki T., Taniuchi T., Nakano H., & Shimizu M., Age, growth and reproduction of the oceanic whitetip shark from the Pacific Ocean. *Fisheries Science* **64**, pp. 14-20 (1998).

[4] Clarke A., & Johnston N.M., Scaling of metabolic rate with body mass and temperature in teleost fish. *Journal Animal Ecology* **68**, pp. 893-905 (1999).

[5] Dowd W.W., Brill R.W., Bushnell P.G., & Musick J.A., Standard and routine metabolic rates of juvenile sandbar sharks (*Carcharhinus plumbeus*) including the effects of body mass and acute temperature change. *Fisheries Bulletin* **104**, pp. 323-331 (2006).

[6] Killen S.S., Atkinson D., & Glazier D.S., The intraspecific scaling of metabolic rate with body mass in fishes depends on lifestyle and temperature. *Ecology Letters* **13**, pp. 184-193 (2010).

[7] Kushmerick M., & Davies R., The chemical energetics of muscle contraction II: The chemistry, efficiency and power of maximally working sartorius muscles. *Proceedings of the Royal Society B* **174**, pp. 315-347 (1969).

[8] Vander A.J., Sherman J.H., & Luciano D.S., *Human physiology*, 4th edition, McGraw-Hill, New York. pp. 91-100 (1985)

[9] Maertens A.P., Triantafyllou M.S., & Yue D.K.P., Efficiency of fish propulsion. *Bioinspiration and Biomimetics* **10**, 046013 (2015).

[10] Iosilevskii G., & Papastamatiou Y.P., Relations between morphology, buoyancy and energetics of requiem sharks. *Royal Society Open Sci*ence **3**, 160406 (2016).

[11] Yates G.T., Hydromechanics of body and caudal fin propulsion. *in* Weihs D., & Webb P.W. (eds.) *Fish biomechanics*, New York: Praeger, pp. 177-213 (1983).

[12] Cheng J.Y, & Blickhan R., *Note on the calculation of propeller efficiency using elongated body theory. Journal Theoretical Biology* **192**, pp. 169-*177* (1994).

[13] Raymer D.P. *Aircraft design: a conceptual approach*, AIAA educational series, Washington DC, pp. 279-281(1992).
